# Somatosensory perception–action binding in Tourette syndrome

**DOI:** 10.1038/s41598-021-92761-4

**Published:** 2021-06-28

**Authors:** Julia Friedrich, Henriette Spaleck, Ronja Schappert, Maximilian Kleimaker, Julius Verrel, Tobias Bäumer, Christian Beste, Alexander Münchau

**Affiliations:** 1 Institute of Systems Motor Science, Center of Brain, Behavior and Metabolism, University of Lübeck, Lübeck, Germany; 2 Cognitive Neurophysiology, Department of Child and Adolescent Psychiatry, Faculty of Medicine, TU Dresden, Dresden, Germany; 3 Department of Neurology, University Medical Center Schleswig-Holstein, Campus Lübeck, Lübeck, Germany; 4 Department of Psychiatry and Psychotherapy, University Medical Center Schleswig-Holstein, Lübeck, Germany

**Keywords:** Cognitive neuroscience, Developmental disorders

## Abstract

It is a common phenomenon that somatosensory sensations can trigger actions to alleviate experienced tension. Such “urges” are particularly relevant in patients with Gilles de la Tourette (GTS) syndrome since they often precede tics, the cardinal feature of this common neurodevelopmental disorder. Altered sensorimotor integration processes in GTS as well as evidence for increased binding of stimulus- and response-related features (“hyper-binding”) in the visual domain suggest enhanced perception–action binding also in the somatosensory modality. In the current study, the Theory of Event Coding (TEC) was used as an overarching cognitive framework to examine somatosensory-motor binding. For this purpose, a somatosensory-motor version of a task measuring stimulus–response binding (S-R task) was tested using electro-tactile stimuli. Contrary to the main hypothesis, there were no group differences in binding effects between GTS patients and healthy controls in the somatosensory-motor paradigm. Behavioral data did not indicate differences in binding between examined groups. These data can be interpreted such that a compensatory “downregulation” of increased somatosensory stimulus saliency, e.g., due to the occurrence of somatosensory urges and hypersensitivity to external stimuli, results in reduced binding with associated motor output, which brings binding to a “normal” level. Therefore, “hyper-binding” in GTS seems to be modality-specific.

## Introduction

It is a common experience that in situations where movements or sounds should be avoided, e.g., in a concert hall, perception of somatosensory stimuli, like a tingling in the nose or scratching in the throat, rises. Such somatosensory sensations often lead to an urge to move or vocalize to reduce experienced tensions. Such urges play a prominent role in patients with Gilles de la Tourette (GTS) syndrome, a common neuropsychiatric neurodevelopmental disorder defined by multiple motor and phonic tics starting before the age of 18 and lasting for more than 1 year^1,2^. In GTS, tics as the cardinal clinical feature are often preceded by localized or diffuse unpleasant sensations including the perception of pressure, hot or cold temperature or tickling, typically causing an urge to move^3,4^. Commonly, urges develop or increase in intensity before the occurrence of tics and are attenuated after tic execution^5^, at least in adult patients. Thus, the experience of urges appears to develop with age, i.e. is more common in adolescents and adults than smaller children with GTS^6^.

In addition to urges, there is also hypersensitivity to specific external stimuli in GTS^7,8^, i.e., it has been shown that GTS patients are more easily distracted and distressed by tactile stimuli^8,9^. This is not explained by an increased perception of low-level stimuli given that basic sensation reflected in perception threshold-measurements is normal in GTS^9,10^. It therefore probably reflects altered perceptual processing. The relevance of somatosensory processing and sensorimotor integration in the pathophysiology of GTS is also corroborated by experimental data. For example, the grip force GTS patients apply to hold an object is higher compared to healthy controls^11,12^. Furthermore, short afferent inhibition tested with peripheral electrical stimulation over the median nerve coupled with transcranial magnetic stimulation (TMS) applied over the motor cortex is reduced in GTS^13,14^. Also, sensory gating has been shown to be reduced resulting in increased sensory input^15^, and there are changes in the structural composition of cortical sensory areas^16–19^.

Against this background, it has been proposed that GTS could be conceptualized as a disorder of altered integration of sensory input and associated motor output^20^. If so, a framework conceiving perception and action as interconnected and interdependent processes should be particularly informative as regards GTS pathophysiology. In this respect, the Theory of Event Coding (TEC)^21^ constituting a framework systematically addressing perception–action coupling, or binding, considering cognitive processes, appears particularly attractive. Due to the universal conceptualization of TEC, it allows to study sensorimotor integration in different modalities including the somatosensory domain^22^. TEC states the formation of so called “object files” that contain stimulus-related details, and “action files” encompassing features related to a specific response^21^. “Event files” establish associations (bindings) between stimulus- and response-related features^23–25^. The network structure of the event file promotes automatic spreading of activation from one network element to another, which is referred to as pattern-completion^25^. Therefore, re-encountering one specific stimulus- or action-related feature is sufficient to activate the entire event file^25–28^. This automatic activation can have advantages, but also disadvantages. In case all stimulus and response features are repeated in consecutive trials, the already established link facilitates correct and fast responses, which is also referred to as “repetition benefits”. However, performance deteriorates if stimulus and response features partially overlap, i.e., if one specific stimulus feature encountered previously requires a different response in the next trial. This phenomenon, called “partial repetition cost”, indicates that the event file needs to be reconfigured to allow correct responding. The degree of partial repetition costs is an indicator of the strength of event file binding^21,23–25,28–31^. Previous studies investigating perception–action binding mainly focused on the visual modality^29,31^. Given the link between premonitory urges and tics and a number of peculiarities of sensorimotor integration outlined above, it has been proposed that the binding of perceptual and action-related features is increased in GTS patients^20,32^. In fact, a recent study of event file coding using a well-established TEC-derived task in the visual domain in adult patients with GTS showed increased binding between stimulus and response features in these patients^33^. In this study, binding of stimulus- and response-related features was investigated using a visual stimulus–response task with different degrees of feature overlap combined with repeating or alternating responses. However, considering the importance of somatosensory and sensorimotor peculiarities in the pathophysiology of GTS outlined above, it appears particularly relevant to examine whether the concept of “hyper-binding” in GTS patients supported by findings in the visual domain^33,34^ also applies to somatosensory-motor integration. To this end, a somatosensory event file binding task introduced recently^22^ (for details please refer to “Materials and methods” section) was used in the present study.

In terms of TEC, it is plausible to assume that in GTS premonitory urge sensations of somatosensory quality triggering specific motor actions to release tension (tics) and these actions are strongly bound. Therefore, in this study where we used an experimental paradigm designed to capture somatosensory-motor binding, it is hypothesized that clinical phenomenology in GTS patients, particularly the urge followed by tic cascade, will be reflected by stronger event file binding in patients compared to controls. Because of reported alterations particularly with respect to somatosensory processing and the strong saliency of the somatosensory modality, we also hypothesize that increased event file binding will not only be present in the somatosensory domain but will be even more pronounced compared to binding in the visual domain. A direct comparison between modalities, however, would require the implementation of the visual paradigm in the current study. Therefore, it is emphasized at this point that the hypothesis regarding the comparison between modalities refers to previous findings in the visual domain as outlined above. This approach is plausible due to a similar structure of the tasks measuring stimulus–response binding as well as a common underlying theoretical framework.

## Results

Clinical characteristics of patients are given in Table 1.

**Table 1:** Clinical characteristics of GTS patients.

Subject	Age, years	Sex	DCI (0–100)	YGTSS total (0–100)	YGTSS tics (0–50)	PUTS (9–36)	YBOCS (0–40)	ADHD-index (0–36)	DSM-ADHD-scale (0–54)
1	23	F	47	46	12	22	0	7	12
2	24	M	52	36	16	22	0	2	4
3	46	F	55	42	12	20	0	15	29
4	45	M	70	20	20	19	0	10	16
5	21	M	41	38	28	24	2	14	26
6	18	M	51	13	13	21	0	2	4
7	26	M	46	42	12	30	0	9	20
8	35	M	61	30	0	25	0	18	27
9	23	F	5	33	25	26	12	24	19
10	19	F	33	19	9	11	0	4	4
11	27	M	78	53	13	16	0	14	13
12	38	F	79	42	22	22	6	26	29
13	35	M	38	48	28	26	14	12	16
14	31	M	40	41	11	21	0	n.a.	n.a.
15	25	F	98	30	10	20	0	26	19
16	29	F	69	62	32	32	24	n.a.	n.a.
17	19	F	48	50	20	n.a.	0	18	18
18	18	M	87	31	31	22	0	4	12
19	27	M	84	18	18	24	2	10	12
20	18	M	41	31	31	14	19	12	13
21	18	M	100	15	15	21	0	17	31
22	46	M	49	47	27	18	2	6	16
23	20	F	58	58	28	21	10	9	12
24	32	M	56	20	20	20	0	9	19
Mean	27.62		57.75	36.04	18.88	21.60	3.79	12.18	16.38

Compilation of clinical characteristics of GTS patients in tabular form. Shown are age, sex, and scores of the respective assessments. Details on each assessment can be found in the text.

*ADHD* attention deficit/hyperactivity disorder, *DCI* diagnostic confidence index, *PUTS* premonitory urge for tic scale, *YBOCS* Yale Brown obsessive compulsive scale, *YGTSS* Yale global tic severity scale, *n.a.* not available.

Four GTS patients were diagnosed with ADHD, three with a depressive disorder, two with OCD. One patient had post-traumatic stress disorder and also had substance abuse in the past, one patient was diagnosed with Asperger syndrome and one had a diagnosis of a psychosomatic disorder. Ten out of 24 patients were on medication during testing. Treatment included aripiprazole (n = 4), tiapride (n = 2), risperidone (n = 1), pimozide (n = 1), biperiden hydrochloride (n = 1) and olanzapine (n = 1). There was no change in medication within at least 4 weeks prior to testing. In both groups, mean IQ was in the normal range. Mean IQ was 103.5 (± 11.8) in GTS patients and 100 (± 8.7) in healthy controls (HC). Two patients and none of the HC were left-handed.

There was no significant group difference regarding age (t(42) = − 0.11, p = 0.916) or IQ (t(42) = − 1.24, p = 0.222). A Pearson’s Chi-Squared test with Yates' continuity correction indicated that there was also no group difference in sex (χ^2^(1) = 0.038, p = 0.845).

### Behavioral data

The Repeated Measures ANOVA conducted for accuracy rates showed a main effect of Response (F(1,42) = 13.6; p < 0.001; η_p_^2^ = 0.042; $B{F}_{01}$ = 0.01) and Group (F(1,42) = 5.8; p = 0.02; η_p_^2^ = 0.087; $B{F}_{01}$ = 0.38). No other main or interaction effect was significant (all F ≤ 3.17; p ≥ 0.082). Descriptive statistics regarding accuracy rates are given in Table 2.

**Table 2:** Descriptive statistics (accuracy rates).

Group	Response alternation and feature (finger) alternation		Response repetition and feature (finger) alternation		Response repetition and feature (finger) repetition		Response alternation and feature (finger) repetition	
	GTS	HC	GTS	HC	GTS	HC	GTS	HC
Mean (percentage correct)	95	98	92	96	91	96	95	97
Standard deviation	5	2	8	4	8	3	6	4

Mean accuracy rates (percentage correct) for each group and condition including respective standard deviations.

The analysis of reaction times revealed a main effect of Response (F(1,42) = 7.21; p = 0.01; η_p_^2^ = 0.005; $B{F}_{01}$ = 0.2), a main effect of Finger compatibility (F(1,42) = 15.94; p < 0.001; η_p_^2^ = 0.004; $B{F}_{01}$ < 0.01) and an interaction of Response × Finger compatibility (F(1,42) = 38.01; p < 0.001; η_p_^2^ = 0.015; $B{F}_{01}<.01$). No other main or interaction effect was significant (all F ≤ 0.74; p ≥ 0.393). Descriptive statistics regarding response times are given in Table 3. The interaction of Response × Finger compatibility signifies stimulus–response binding showing that event file binding effects in the somatosensory domain were present in the task used^31^. However, the lack of interaction with the factor Group (F(1,42) = 0.15; p = 0.7; η_p_^2^ < 0.001; $B{F}_{01}$ = 6.13) shows that stimulus–response binding did not differ between HC and GTS patients. The Bayesian factor also indicates support in favor of the null hypothesis^35^.

**Table 3:** Descriptive statistics (response times).

Group	Response alternation and feature (finger) alternation		Response repetition and feature (finger) alternation		Response repetition and feature (finger) repetition		Response alternation and feature (finger) repetition	
	GTS	HC	GTS	HC	GTS	HC	GTS	HC
Mean (in ms)	557	558	587	594	556	561	570	565
Standard deviation	94	75	98	88	91	72	96	80

Mean response times in milliseconds (ms) for each group and condition including respective standard deviations.

Post-hoc paired t-tests performed across all participants showed that stimulating a finger repeatedly versus stimulating alternating fingers led to significantly different reaction times for repeated responses (t(42) = 7.30; p < 0.001; $B{F}_{01}<.01$) but not for alternating responses (t(42) =  − 2.27; p = 0.154; $B{F}_{01}=.54$). For response repetition, responses were faster when the same finger was stimulated (558 ms ± 82) than when stimulated fingers alternated (590 ms ± 92). Post-hoc paired t-tests revealed that response repetition and alternation differed when the finger was alternated (t(42) = − 5.91; p < 0.001; $B{F}_{01}<.01$) but these conditions did not differ when stimulation was repeated at the same finger (t(42) = 1.68; p = 0.581; $B{F}_{01}=1.79$). In case of finger alternation, responses were faster when the response was alternated (558 ms ± 84) than when it was repeated (590 ms ± 92).

**Figure 1:**
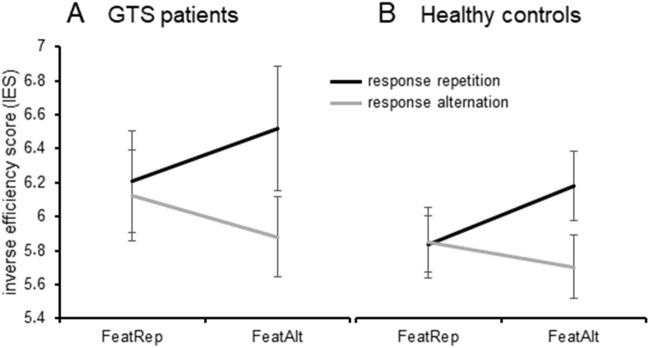
Behavioral data (inverse efficiency score) demonstrating the interaction of Response × Finger compatibility for GTS patients (**A**) and healthy controls (**B**). The IES is illustrated separately for GTS patients (**A**) and healthy controls (**B**) for response repetition and response alternation in the feature (finger) repetition (FeatRep) condition and the feature (finger) alternation (FeatAlt) condition. Error bars represent the standard error of the mean (SEM).

The results regarding the interaction of Response × Finger compatibility differed between accuracy rates and reaction times. Therefore, we computed the inverse efficiency score (IES) to account for a speed-accuracy trade-off^36^. Superior performance leads to lower IES values. The repeated measures ANOVA showed a main effect of Response (F(1,42) = 11.26; p = 0.002; η_p_^2^ = 0.015; $B{F}_{01}$ = 0.04) and an interaction of Response × Finger compatibility (F(1,42) = 18.02; p < 0.001; η_p_^2^ = 0.011; $B{F}_{01}$ < 0.01). Behavioral interaction effects are displayed for both groups separately in Fig. 1. No further main or interaction effect was significant (all F ≤ 3.89; p ≥ 0.055).

There was no significant three-way interaction of Group × Response × Finger compatibility (F(1,42) = 0.071; p = 0.791; η_p_^2^ < 0.001; $B{F}_{01}$ = 6.39). The Bayesian factor also indicates support in favor of the null hypothesis.

Post-hoc paired t-tests performed across all participants showed that repetition and alternation of finger stimulation differed when the response was the same (t(42) = 4.67; p < 0.001; $B{F}_{01}$ < 0.01) and when the response was alternated (t(42) = − 2.75; p = 0.046; $B{F}_{01}$ = 0.13). In case of repeated responses, performance was better when the finger was repeatedly stimulated compared to alternating the stimulated finger, but for alternating responses, performance was worse when the finger was repeatedly stimulated compared to the condition when finger stimulation was alternated. Post-hoc paired t-tests also showed that response repetition and alternation conditions differed when the stimulated finger alternated (t(42) =  − 5.18; p < 0.001; $B{F}_{01}$ < 0.01), but did not differ when the same finger was stimulated repeatedly (t(42) = − 0.34; p = 1; $B{F}_{01}$ = 6).

To investigate a possible relationship between clinical characteristics of the GTS group and binding effects at the behavioral level, Pearson’s correlations were conducted. Rebinding cost, that is, the difference between binding-incompatible and binding-compatible [(response repetition, feature alternation) + (response alternation, feature repetition)] − [(response repetition, feature repetition) + (response alternation, feature alternation)] conditions were calculated for reaction time and accuracy. These measures were then correlated with the YGTSS and PUTS. There was a correlation between the total tic severity score and the rebinding costs for the reaction time (r(21) = 0.42; p = 0.044). None of the other correlations between the clinical and behavioral binding measures were significant (r ≥ − 0.22; p ≥ 0.144).

## Discussion

The current study aimed at investigating somatosensory-motor event file binding in GTS patients compared to healthy controls. Given the presence of prominent sensory phenomena in GTS, i.e., urges preceding tics and increased sensitivity to external stimuli^7,8^, abnormalities of sensorimotor integration processes^11,12^, as well as documented increased binding of stimulus and response features in the visual domain in these patients^33^, it was hypothesized that binding of sensory input and associated motor output would also be increased in the somatosensory modality, probably over and above “hyper-binding” in the visual domain. Particularly the occurrence of urges that can be triggered by somatosensory sensations^3^ supported the assumption of increased binding in the somatosensory modality. Again, it should be emphasized that visual binding effects were not tested in the current study, so that a direct comparison between the modalities cannot be made. Nonetheless, due to a similar task structure and a common underlying theoretical framework, hypotheses regarding the somatosensory modality are derived from the results in the visual domain. Here, we tested a novel recently established somatosensory-motor task^22^ in the context of TEC^21^ providing a suitable theoretical framework to investigate alterations in somatosensory-motor binding since it systematically addresses perception–action coupling.

Contrary to our main hypothesis, behavioral results (i.e., accuracy rates, reaction times or the inverse efficiency score) revealed no indication of increased binding in the somatosensory-motor domain in GTS patients compared to healthy controls. The finding that there was no significant interaction effect of Group × Response × Finger compatibility evident for behavioral data was supported by Bayesian analyses providing evidence for a lack of effect. Therefore, the likelihood of a false negative finding is very low. Although somatosensory rather than visual processing seems to be particularly altered in GTS, “hyper-binding” could be demonstrated experimentally when testing visuo-motor^33^ but not when testing somatosensory-motor processing. At first sight and from a clinical perspective, this finding appears to be counterintuitive.

If perception–action “hyper-binding” is a core abnormality in GTS, which is suggested by the correlation between the strength of perception–action binding and the frequency of tics, i.e., the cardinal feature of GTS in the study of Kleimaker et al. addressing visuo-motor event file binding^33^, a lack of “hyper-binding”, i.e. relatively attenuated binding in the somatosensory compared to the visual domain in this study, might be explained by abnormalities of processing of somatosensory stimuli.

First, differences of perceptual thresholds need to be considered. However, using standardized quantitative sensory testing (QST) examining sensory parameters including thermal, mechanical/tactile and pain stimuli, thresholds have been shown to be normal in adult patients with GTS^9,10^. Also, there was no relation between QST parameters and premonitory urges in these patients^10^. Thus, mechanisms other than stimulus detection including central somatosensory-motor processing and/or aberrant interoceptive awareness might play a role. For instance, it has been shown that short afferent inhibition tested by delivering electric pulses to the median nerve at the wrist is decreased in GTS^13,14^. This can be interpreted as a reduction of sensorimotor processing, which is further supported by grip force experiments showing that GTS patients use higher grip force to hold an object with defined weight compared to healthy controls^11^ and findings of diminished sensory gating^15^. These data can be interpreted such that presumably increased activity in the somatosensory system in GTS as evidenced by urges^5^ and hypersensitivity to external stimuli^7,8^ leads to central adaptation, i.e. inhibition of sensorimotor processes with the effect that the salience of somatosensory stimuli is attenuated. As a consequence, it is plausible that also the association (i.e., binding) of somatosensory input with motor output is weakened resulting in a “normal” level of binding instead of “hyper-binding”. Alternatively, it is also possible that increased sensory feedback at a cortical level leads to superior motor control due to enhanced communication with motor areas^15^. It can be assumed that this allows increased control over motor output so that it is easier to more flexibly adjust to the currently presented stimulus–response association. Supporting this, it has already been suggested that constant tic suppression may result in a general benefit with regard to the self-regulation of motor responses (i.e. enhanced cognitive control) as indicated by lower switch costs in children with less tic severity^37^. This can also be a reason why task performance between patients and controls did not differ. Supporting this assumption, we also found a positive correlation between total tic severity as indicated by the YGTSS and the rebinding costs in the GTS group suggesting that less successful tic suppression impairs the ability to flexibly adjust stimulus–response binding. In case a sample of patients encompasses a majority of individuals capable of suppressing their tics, this could result in task performance similar to healthy controls. Further studies directly investigating the impact of the ability to suppress tics on binding effects (i.e. partial repetition costs) might resolve this issue.

“Hyper-binding” in GTS seems to be rather modality-specific. This interpretation is also supported by an fMRI study where participants had to withhold a prepared finger movement for a variable time until a stimulus instructed them to either execute or inhibit it^38^. In GTS, functional brain activation was decreased in sensorimotor cortical areas during movement execution, which was interpreted as an adaptive inhibitory reorganization in fronto-parietal brain networks. It is also corroborated by a study investigating echophenomena in GTS^39^. In line with the notion of an increased sensitivity to certain external stimuli, it is well known and has experimentally been shown that GTS patients have an increased tendency to imitate automatically what they hear or see, i.e., they show echophenomena (echolalia and echopraxia)^40,41^, which suggests increased automatic activation of motor output triggered by perceptual input. However, the study found a stronger adaptive inhibition of responses to biological stimuli in GTS compared to healthy controls demonstrating that there is an alteration of perception–action binding in the way that automatic activation of motor output triggered by perceptual input is downregulated in patients in order to prevent unwanted movement. What emerges is a general theme of presumably disease-related over-activity of certain perceptual processes and concomitant increased inhibitory activity of these processes, probably as an adaption. This assumption of a “downregulation” of the supposedly accentuated modality resulting in reduced saliency of certain stimuli including somatosensory stimuli is further supported by a study demonstrating reduced interoceptive awareness in GTS patients^42^. Interoceptive awareness was associated with stronger urges and higher tic severity. Reduced interoceptive awareness could therefore be interpreted as “collateral damage” due to the inhibition of the somatosensory system, or in other words, the decreased saliency of somatosensory input might also affect the ability to be interoceptively aware. Alternatively, attenuated interoceptive awareness might also affect somatosensory processes, e.g., by reducing attention paid to body sensations.

Taken together, the extent of “hyper-binding” in GTS is probably modality dependent. It appears that “hyper-binding” is attenuated by compensatory downregulation in the somatosensory modality, but more pronounced in the visual modality, where no attenuation occurs.

To sum up, the present study addressed differences in somatosensory-motor event file binding in GTS patients compared to healthy controls. Surprisingly, behavioral data did not reveal group differences in binding effects indicating a lack of “hyper-binding” in the somatosensory domain as compared to results from the visual domain^33^. However, it should be kept in mind that visual- and somatosensory-motor paradigms to measure event file binding remarkably differ so that a direct comparison cannot be made without considering modality differences. Due to its universal conceptualization, TEC provides a useful tool to investigate sensorimotor integration in different modalities. We speculate that a compensatory “downregulation” of somatosensory stimulus saliency as a response to the presence of urges and hypersensitivity to somatosensory stimuli results in a weakened binding with associated motor output, so that “hyper-binding” is no longer evident in the somatosensory modality.

## Materials and methods

### Participants

The data of N = 24 GTS patients (15 males and 9 females, mean age 27.62 years ± 1.82 SEM, range 18–46 years) was analyzed.

Patients were recruited from the specialized GTS outpatient clinic in the Center for Integrative Psychiatry at the University Medical Center Schleswig–Holstein, Campus Lübeck. They were diagnosed according to DSM-5 criteria^2^. Clinical assessment was performed by experienced neurologists. To quantify tic severity and premonitory urges, the Yale Global Tic Severity Scale (YGTSS)^43^ and the Premonitory Urge for Tics Scale (PUTS) were used^44^. The Diagnostic Confidence Index (DCI) was utilized to specify the lifetime likelihood of a diagnosis of GTS^45^. The Mini International Neuropsychiatric Interview (M.I.N.I.) was used to collect information on psychiatric comorbidities^46^. Attention deficit hyperactivity disorder (ADHD) was assessed according to DSM-5. OCD (obsessive–compulsive disorder) symptoms were measured with the Yale Brown Obsessive Compulsive Scale (YBOCS)^47^ and ADHD symptoms using the ADHD-Index and the DSM-ADHD Scale of the German version of the Conners Adult ADHD Rating Scale (CAARS)^48^. Handedness was registered using the Edinburgh Handedness Inventory^49^. For IQ assessment, the German version of the fourth edition of the Wechsler Adult Intelligence Scale (WAIS) was employed^50^.

Furthermore, the data of N = 20 healthy controls was analyzed (11 males and 9 females, mean age 26.75 years ± 1.63 SEM, range 18–51 years). Participants were recruited via e-mail distribution lists at the University of Lübeck, posters around the city and on campus. Furthermore, adverts were placed online at a digital marketplace.

An interview with respect to psychiatric and neurological disorders was also carried out in healthy controls. The Mini International Neuropsychiatric Interview (M.I.N.I) was used to assess psychiatric comorbidities. None of the healthy participants had psychiatric symptoms at the time of testing. Furthermore, all participants were required to have no history of manual disturbances (e.g. numbness or tingling). Participants with an IQ below 80 were excluded. Healthy controls were excluded in case of history of tics or neurologic or psychiatric disorders.

Participants gave written informed consent to participate in the study in accordance with the Declaration of Helsinki (1964). The Ethics Committee of the University of Lübeck approved the study (reference number 17-157).

### Procedure and task

We used the somatosensory version of the original visual stimulus–response (S-R) paradigm^23^. Instead of visual stimuli, electro-tactile stimuli (bipolar electrical pulses, 0.2 ms duration, 300 V) were used to examine somatosensory-motor binding. Electro-tactile stimulation was delivered via eight disposable surface adhesive electrodes. Electrodes were attached in pairs (with a distance of about 1 cm from each other) at each site, i.e., the back of both hands as well as the palmar sides of the right thumb and right little finger. We opted for thumb and little finger because these are easier to differentiate than adjacent fingers. During the experiment, we applied two or four pulses to the thumb or little finger. Thus, in the double pulse condition, two pulses were given at a frequency of 6 Hz, so that the stimulation duration was 166 ms. The quadruple pulse consisted of four pulses at 12 Hz resulting in a 250 ms stimulation period.

**Figure 2:**
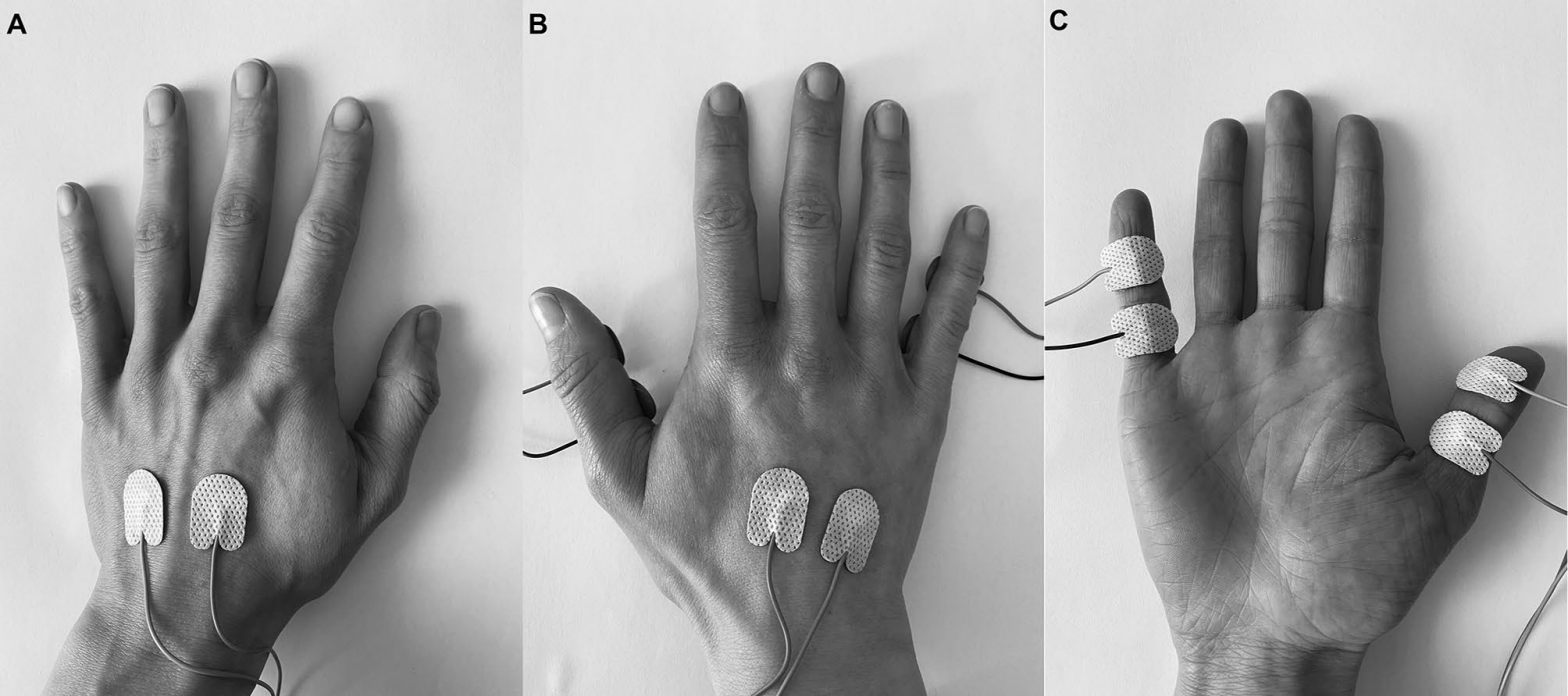
Illustration of the experimental setup (electrode placement). Stimulation was applied via adhesive surface electrodes attached to (**A**) the back of the left hand, (**B**) the back of the right hand and (**C**) the palmar side of the thumb and little finger of the right hand. Responses were given by pressing the left or right control key with the respective index finger.

An ISIS Neurostimulator (Inomed, Emmendingen, Germany; https://www.inomed.de/) was used to generate electro-tactile stimulation. The experimental software necessary to control the device was developed in Python 2.7 employing the “expyriment toolbox”^51^. The experimental setup (electrode placement) is illustrated in Fig. 2.

**Figure 3:**
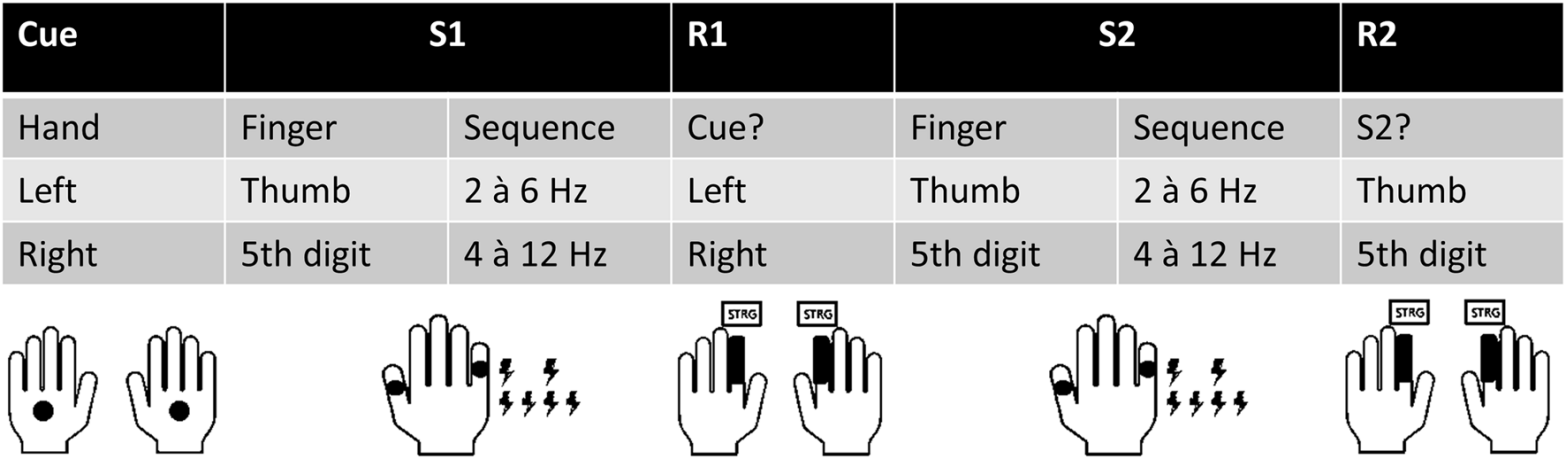
Schematic illustration of the experimental procedure. For clarity, one black dot represents a pair of electrodes. Electro-tactile stimuli were either applied to the thumb or little finger (5th digit) using a double (2 à 6 Hz) or quadruple (4 à 12 Hz) pulse. Details concerning stimulus and response timing can be found in “Materials and methods” section.

At the beginning of each trial, a cue consisting of a single electrical pulse was presented at the back of either the left or right hand. After a time period of 2500 ms, stimulus 1 (S1), encompassing either two or four electrical pulses, was applied to the thumb or the little finger of the right hand. After S1 presentation, subjects had to indicate where the cue was applied (back of the left or the right hand) by pressing the left control key with their left index finger or the right control key with their right index finger independently from any features of S1. In case participants responded incorrectly or too slowly, i.e., exceeded the time period of 700 ms after S1 onset, the German word for repetition (“Wiederholung”) was shown for 500 ms. The trial was repeated a maximum of three times, or then discarded. Subsequently, stimulus 2 (S2) was applied to the right thumb or right little finger 2500 ms after S1 offset. Participants were then asked to indicate whether S2 was delivered to the thumb by pressing the left control key with their left index finger or whether the little finger was stimulated by pressing the right control key with their right index finger. For responses to be registered, they had to be given within a time window of 2000 ms after S2 offset. Inter-trial intervals were jittered between 1500 to 2000 ms. This experimental setup resulted in different compatibility conditions. If pulse sequence and stimulation site corresponded between S1 and S2, all features were compatible. There was no compatibility if different pulse sequences and stimulation sites were used. One feature was compatible in case either the same pulse sequence was applied, or the same finger was stimulated. Furthermore, the responses to the cue at the time of S1 presentation and to S2 could either differ (response alternation) or not (response repetition). Consequently, repetition benefits and repetition costs could be examined. Across the experiment, each condition was presented equally often. It is typically observed that during the response repetition condition, task performance improves the more stimulus features are repeated (i.e., are compatible) (repetition benefits). In case of response alternation, a repetition of stimulus features impairs performance (repetition costs)^23,52^. A complete overlap or non-overlap of stimulus and response features results in superior performance compared to the overlap of some features which is referred to as “partial-repetition costs”^25,28^. “Finger” (stimulation site thumb or little finger) and “sequence” (a number of two or four pulses) constituted the different stimulus dimensions since these stimulus features are clearly separable. A schematic illustration of the experimental procedure is shown in Fig. 3.

384 trials were presented, divided into six blocks, with equal frequency of each condition within these blocks. To avoid predictability of stimulus sequence or stimulation site, conditions were presented randomly. A practice block with 16 trials was conducted before the experiment. Each trial was announced by the German expression for next trial (“Nächster Durchgang”) presented for 500 ms.

### Statistical analysis

Behavioral data, i.e., accuracy rate and response time in correct trials as well as the inverse efficiency score constituting the ratio of these two measures (i.e. accuracy divided by mean response time) to account for a speed-accuracy trade-off^36,53^, was analyzed using Repeated Measures analyses of variances (ANOVAs). The reaction time data were summarized per subject and condition using the median (not the mean) in order to reduce the influence of outliers. To this end, two within-subject factors were defined. The factor Finger compatibility defined whether (S1 and S2) stimulation was delivered to the same finger twice (finger repetition) or to different fingers (finger alternation). The factor Response was used to describe whether the same (response repetition) or different responses (response alternation) were required in reaction to S1 and S2. The factor Group (GTS patient or healthy control) was set as between-subject factor. Based on previous results^22^ revealing no effect of the factor Sequence (repetition or alternation of pulse sequence), it was not included in the current analysis. All tests were Greenhouse–Geisser corrected and all post-hoc tests Bonferroni corrected. The ANOVAs as well as the post-hoc tests were carried out in JASP. To further examine main and especially interaction effects, we conducted a Bayesian analysis applying the method by Masson^54^. We report $B{F}_{01}$, which indicates the Bayesian factor in favor of the null hypothesis over the alternative hypothesis. Small values indicate support for ${H}_{1}$, large values for ${H}_{0}$ and a value of one shows equal support for the null and the alternative hypothesis^35^.

## Data Availability

The datasets generated and/or analyzed during the current study are available from the corresponding author on reasonable request.
